# Assessing the impact of AGS-004, a dendritic cell-based immunotherapy, and vorinostat on persistent HIV-1 Infection

**DOI:** 10.1038/s41598-020-61878-3

**Published:** 2020-03-20

**Authors:** Cynthia L. Gay, Joann D. Kuruc, Shane D. Falcinelli, Joanna A. Warren, Sarah A. Reifeis, Jennifer L. Kirchherr, Katherine S. James, Morgan G. Dewey, Alyson Helms, Brigitte Allard, Erin Stuelke, Alicia Gamble, Ana Plachco, Robert J. Gorelick, Joseph J. Eron, Michael Hudgens, Carolina Garrido, Nilu Goonetilleke, Mark A. DeBenedette, Irina Y. Tcherepanova, Charles A. Nicolette, Nancie M. Archin, David M. Margolis

**Affiliations:** 1 University of North Carolina HIV Cure Center, UNC Institute of Global Health and Infectious Diseases, Chapel Hill, NC, United States; 2 Department of Medicine, UNC Chapel Hill School of Medicine, Chapel Hill, NC, United States; 3 Department of Microbiology and Immunology, UNC Chapel Hill School of Medicine, Chapel Hill, NC United States; 4 Department of Biostatistics, UNC Chapel Hill Gillings School of Public Health, Chapel Hill, NC United States; 5 Department of Epidemiology, UNC Chapel Hill Gillings School of Public Health, Chapel Hill, NC United States; 6 AIDS and Cancer Virus Program, Frederick National Laboratory for Cancer Research, Frederick, MD United States; 7 Argos Therapeutics, Durham, North Carolina USA

**Keywords:** Immunization, Viral reservoirs, HIV infections, Translational research

## Abstract

Approaches to deplete persistent HIV infection are needed. We investigated the combined impact of the latency reversing agent vorinostat (VOR) and AGS-004, an autologous dendritic cell immunotherapeutic, on the HIV reservoir. HIV+, stably treated participants in whom resting CD4^+^ T cell-associated HIV RNA (rca-RNA) increased after VOR exposure *ex vivo* and *in vivo* received 4 doses of AGS-004 every 3 weeks, followed by VOR every 72 hours for 30 days, and then the cycle repeated. Change in VOR-responsive host gene expression, HIV-specific T cell responses, low-level HIV viremia, rca-RNA, and the frequency of resting CD4^+^ T-cell infection (RCI) was measured at baseline and after each cycle. No serious treatment-related adverse events were observed among five participants. As predicted, VOR-responsive host genes responded uniformly to VOR dosing. Following cycles of AGS-004 and VOR, rca-RNA decreased significantly in only two participants, with a significant decrease in SCA observed in one of these participants. However, unlike other cohorts dosed with AGS-004, no uniform increase in HIV-specific immune responses following vaccination was observed. Finally, no reproducible decline of RCI, defined as a decrease of >50%, was observed. AGS-004 and VOR were safe and well-tolerated, but no substantial impact on RCI was measured. In contrast to previous clinical data, AGS-004 did not induce HIV-specific immune responses greater than those measured at baseline. More efficacious antiviral immune interventions, perhaps paired with more effective latency reversal, must be developed to clear persistent HIV infection.

## Introduction

Life-long antiretroviral therapy (ART) is required to prevent rebound of viremia and return of disease, due to the persistence of long-lived viral reservoirs. Multiple mechanisms contribute to the complexity of latent and persistent HIV infection^1^. One of the best studied drivers of HIV latency is epigenetic control of integrated proviral DNA^2^. Lysine acetylation of histone tails by histone acetyl transferases is believed to result the neutralization of basic charges of core histones, leading to destabilization of DNA/histone interactions, increased accessibility of transcription factors to the HIV promoter (LTR), and transcription initiation. Deacetylation of lysine residues on histone tails by histone deacetylases (HDACs) is associated with decreased access of positive transcription factors to the LTR, and recruitment of histone-modifying and chromatin complexes implicated in transcriptional repression. HDAC inhibition antagonizes repression of HIV transcription, and has been investigated as a tool to reverse HIV latency in humans^3–7^. While administration of HDAC inhibitors (HDACi) *in vivo* increased HIV transcription in each of these studies, a corresponding depletion of persistent infection has not been observed. Reversal of HIV latency at the transcriptional level may not deplete persistent infection because a) the effect is insufficiently broad or too transient to impact the reservoir, and/or b) immune mechanisms are of insufficient frequency or function to clear persistently infected cells following latency reversal, even in ART-treated individuals.

Exposure to the HDAC inhibitor vorinostat (VOR) induces HIV antigen expression sufficiently to allow viral clearance *in vitro*^8,9^. Four studies^3,5–7^ have demonstrated effective latency reversal *in vivo* at the level of cell-associated HIV RNA production following an initial dose of vorinostat (two studies), panobinostat, or romidepsin (one study each). Vorinostat has been shown to effectively and repeatedly reverse latency *in vivo* at the level of viral gene expression when given every 72 hours^4^. Therefore, we sought to determine whether the addition of an immunotherapy to the administration of a latency reversing agent (LRA) would clear reactivated, formerly latently HIV-infected cells, leading to a decrease of the HIV reservoir. AGS-004 is a dendritic cell (DC)-based immunotherapy consisting of matured autologous DCs co-electroporated with *in vitro* transcribed ribonucleic acid (RNA) encoding autologous HIV antigens (*gag*, *vpr*, *rev*, and n*ef*) plus synthetically derived cluster of differentiation 40 ligand (CD40L) RNA to achieve DC functionality^10^. This vaccine was reported to induce HIV-specific effector/memory CD8 T-cell responses in HIV-infected individuals who had initiated ART during acute or chronic infection^11–13^.

In a prior open-label, single arm sub-study, AGS-004 was administered monthly to suppressed participants who started ART during acute HIV infection^13^. Participants demonstrating increased immune response after vaccination were eligible for analytic treatment interruption (ATI). The frequency of resting CD4^+^ T-cell infection (RCI) was measured by quantitative viral outgrowth assay. All participants underwent ATI with rebound viremia at a median of 29 days. AGS-00 before and after vaccination. induced a ≥2-fold increase from baseline in the number of multifunctional HIV-1 specific CD28^+^/CD45RA^−^ CD8^+^ memory cytotoxic T-lymphocytes (CTLs) in all six participants, but did not permit sustained ART interruption. However, greater expansion of CD28^−^/CCR7^−^/CD45RA^−^ CD8^+^ effector T cell responses correlated with a longer time to viral rebound. In this study we investigated the impact of VOR combined with AGS-004, on persistent HIV infection.

## Results

### Clinical outcome of VOR/AGS-004 combination

Twelve ART-treated, stably aviremic participants were initially enrolled in this step-wise study. Informed consent was obtained from all patients prior to study enrollment. Baseline ART was maintained throughout the study. Given the theoretical risks of vorinostat, and the low likelihood of clinical benefit in this study, we first confirmed *ex vivo* exposure to VOR resulted in a measurable increase in rca-RNA in the resting CD4+ T cells of participants from a donation provided after screening. Participants who had a measurable response to VOR *ex vivo* as measured by a significant increase in rca-RNA in resting CD4^+^ T cells progressed to receive a single 400 mg oral dose of VOR. If a significant increase in rca-RNA was measured, then response following two paired *in vivo* doses of VOR given at a 72 hr interval was assayed, to confirm that a 72 hr dosing interval was reproducibly effective at inducing rca-RNA *in vivo*, as previously documented^4^. If a significant increase in rca-RNA was observed after the paired dose, then the participants went on to receive the combination of VOR and AGS-004.

Six of the 12 participants did not progress forward to receiving the single VOR dose either because of an absence of significant *ex-vivo* response to VOR (n = 5, data not shown) or withdrawal of consent due to personal circumstances unrelated to the study (n = 1). All 6 remaining participants received a single and then paired *in vivo* doses of VOR. We did not observe a significant increase in HIV RNA from the resting CD4+ T cells after administration of the *in vivo* paired VOR doses in one participant (VV-05), and thus per protocol this participant did not progress to receive further study treatments (data not shown).

Five participants, all male and white, received AGS-004 intradermal injections, and each dose consisted of 3 injections. The study was closed to accrual thereafter, as manufacturer of the AGS-004 vaccine could no longer produce vaccine for the study. The median age was 50 years [range 29–62]. Viremia in all participants was suppressed (<50 copies/ml) for at least 5.1 years [range 5.1–7.8] with a median baseline CD4^+^ count of 408 cells/µl [range 382–746] and median CD4 nadir of 166 cells/µl [range 81–403 l] at enrollment (Table 1).

**Table 1:** Participant characteristics.

PID	Race	Age (years)**	CD4 count(cells/ul) at enrollment	Nadir CD4^Ψ^	Suppression (years)	ART Regimen**	Status at ART Initiation*
VV-01	Caucasian	49.6	542	403	8	FTC, EFV, TDF	AHI
VV-02	Caucasian	54.4	682	81	6.9	RTV, TDF, FTC, NVP, fAPV	CHI
VV-03	Caucasian	30.2	382	354	5.8	TDF, FTC, ETR	CHI
VV-04	Caucasian	54.3	408	157	6.1	EVG, COBI, FTC, TDF	CHI
VV-06	Caucasian	28.4	701	166	4.9	DRV, RTV, TDF, FTC	CHI
**Median**	—	**49.6**	**542**	**166**	**6.1**	—	—

*AHI: acute HIV Infection; CHI: chronic HIV infection.

**At baseline.

^Ψ^Pre-Art.

**Figure 1:**
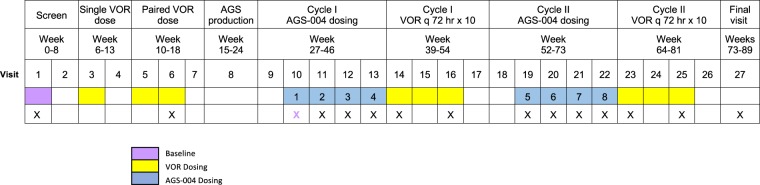
AGS-004–009 Study Design. A) Overall study design. Resting CD4^+^ T cell-associated HIV *gag* RNA was measured at baseline, after the single dose, the paired dose and the first and second cycle of VOR plus AGS-004. RCI was measured at baseline, and after the first and second cycle of VOR+ AGS-004. X details immune monitoring (IM) sample collection to determine immune responses by multi-color flow cytometry. X denotes the timepoints of blood draw collection. The purple X (Visit 10) represents the time point before the administration of AGS-004 and serves as the baseline prior to immunological measurements (unless otherwise noted in a given figure).

Participants received 4 consecutive doses of AGS-004 administered every 3 weeks over 12 weeks. Seven to ten days following the last dose of AGS-004, participants initiated ten consecutive doses of VOR at 72 hr intervals. The cycle of 4 doses of AGS-004 followed by 10 doses of VOR was repeated within 10 weeks of completing the first cycle, for a total of eight AGS-004 doses and 20 doses of VOR over approximately 10 months (Fig. 1). Both AGS-004 and VOR were well tolerated by all participants with only mild AGS-004 site injection reactions, and possible VOR-associated mild, transient GI symptoms No symptoms approached grade I toxicity levels, and all resolved without treatment.

### Expression of HDACi responsive genes confirm *in vivo* bioactivity of VOR

**Figure 2:**
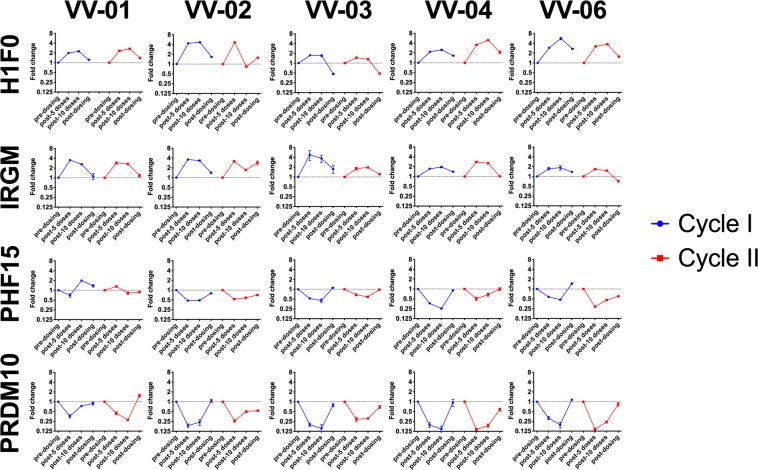
Vorinostat dosing resulted in induction of HDACi-responsive host genes in PBMCs. PBMCs were collected immediately prior to the 1st VOR dose, after the 5th and 10th q72h dose, and 7–10 days after the 10th dose during cycle I and II. Expression of HDACi-responsive host genes was assessed relative to the pre-dose time point for each cycle. Error bars represent standard deviation of the mean of triplicate cDNA preparations, each conducted in duplicate qPCR reactions.

To confirm pharmacologic effect of VOR on cellular targets *in vivo*, we evaluated the expression of several genes with predictable behavior in response to HDAC inhibition *in vitro*. H1F0 and IRGM are upregulated and PHF15 and PRDM10 are downregulated in bulk PBMCs as well as resting CD4^+^ T cells following exposure to HDACi^14^. Across the 5 participants, we observed a pattern of gene modulation consistent with that described in *ex vivo* experiments and after a single *in vivo* VOR dose. H1F0 and IRGM were upregulated during q72 hour VOR dosing relative to baseline and returned to baseline levels after the cessation of dosing. Similarly, PHF15 and PRDM10 were downregulated during VOR dosing and returned to baseline expression levels after completion of dosing (Fig. 2). There were a few departures from this trend in some participants where certain genes were not modulated or modulated in the opposite direction from predicted (VV-01, PHF15; VV-02, H1F0 and IRGM); likely due to the multifactorial nature of transcriptional programming *in vivo* and variation associated with clinical sampling. Overall, however, these data demonstrate that the pharmacological exposure to VOR in this study was sufficient to repeatedly alter host gene transcription over time.

### Minimal impact on the latent HIV reservoir by VOR and AGS-004

It is hypothesized that therapies to disrupt virological latency, in combination with the use of vaccines or immunotherapies to assist in clearance of cells expressing HIV antigens, will be required to deplete the HIV reservoir. To that end we employed a dosing regimen of VOR that can repeatedly reverse latency *in vivo*^4^ together with an immunotherapeutic shown to generate HIV-specific immune responses^10^. We used several approaches to assess the impact of combined immunotherapy and latency disruption on the HIV reservoir. rca-RNA was measured at baseline, and after the first and second cycle of combined therapy. Low-level viremia was measured by a single-copy assay (SCA) at baseline, after the first series of AGS-004 administrations, after the first and second cycle of VOR and AGS-004, and 7–10 days after the last dose of VOR. Finally, we measured the frequency of resting CD4^+^ T-cell infection by the quantitative viral outgrowth assay at the same time points as rca-RNA measurements. While the small sample size is a limitation of this study, we have previously identified a 6-fold decline in RCI measurement as a reliable threshold to assess the efficacy of anti-latency interventions in an individual, making it possible to capture a significant decrease in RCI even with a small sample size^15^.

**Figure 3:**
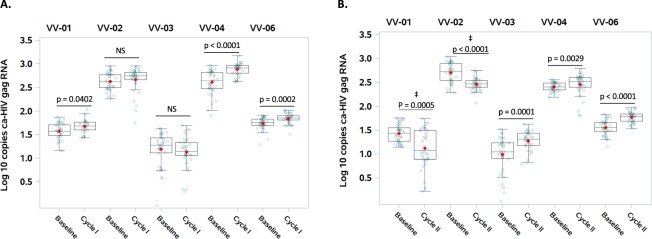
The effect of AGS-004 and VOR on HIV gag ca-RNA expression. Cell-associated HIV RNA was measured from pools of 24–36 million resting CD4+ T cells isolated from participants at baseline and after the first and second cycle of VOR+ AGS-004. Wilcoxon Two-Sample Test was used to assess significant differences in HIV Gag caRNA between baseline and cycle I (**A**) and baseline and Cycle II (**B**) samples. Mean and SD is shown with corresponding p values. ^‡^Indicates a decrease in caRNA following cycle II of AGS-004 +VOR with corresponding p values.

We observed quantitative increases in HIV Gag rca-RNA after the first and second therapy cycle in two of five participants (VV-04 and VV-06), and after the second therapy cycle in a third participant (VV-03), which could be due to VOR induction in the absence of sufficient cell clearance, or simply assay variance. However, in VV-01 and VV-02 we observed increases in rca-RNA after the first therapy cycle but a significant decrease in HIV Gag rca-RNA following the second round AGS-004 and VOR administration (Fig. 3). We observed a significant decrease in low-level viremia as measured by SCA (Table S1) for VV-02 (4.3 copies/ml at entry, and less than 0.5 copies/ml in three assays between visit 14 and 25), but viremia in other participants was either <1.0 or 1.1 copies/ml at all timepoints (VV-01, VV-03, VV-04), or intermittently detected from <1.0 to 84 copies/ml without a clear pattern (VV-06). However, the observed changes in rca-RNA and low-level viremia was not associated with a corresponding depletion of the replication competent reservoir as we failed to observe a significant decrease in RCI in all the participants (Table S2). Not unexpectedly, HIV DNA with resting CD4+ T cells was also stable (Fig. S1).

### *Ex-vivo* T cell immune reactivity to AGS-004

T cell responses to HIV proteins were measured longitudinally by *ex vivo* IFN-γ ELISpot in each participant at time points before and after VOR and AGS-004 dosing. T cell responses were measured against both, proteins within the AGS-004 vaccine (Gag, Nef-Rev-Vpr), and others not in the vaccine (Env, Pol, Tat-Vif-Vpu). In VV-01, VV-02, VV-04, and VV-05, T cell responses to all HIV proteins, both those included or not included in the vaccine, were stable across time points. Less than a 1.5-fold change was observed from baseline in the frequency of HIV-specific T cell responses, whether total, or targeting proteins within or outside of the vaccine’s immunogens (Fig. S2). In VV-03, the summed HIV-specific T cell response increased ~1.5 fold over the course of the study. After the first cycle of AGS-004 dosing, summed HIV-specific T cell response increased from 1,434 SFU/10^6^cells at visit 2 to 2,121 SFU/10^6^cells at visit 16. The summed HIV-specific T cell response again increased after cycle 2 from 1,390 SFU/10^6^cells at visit 20 to 2,250 SFU/10^6^cells at visit 25. Importantly, the increases to the summed HIV-specific T cell response were driven by increases in T cells targeting HIV proteins found in the vaccine (Gag, Nef/Rev/Vpr subpool, 1093 SFU/10^6^cells at visit 2 to 1,751 SFU/10^6^cells at visit 16, and 1,105 SFU/10^6^cells at visit 20 to 1,951 SFU/10^6^cells at visit 25), and not to proteins outside of AGS-004 (Pol, Env, Tat/Vif/Vpu subpool, 341 SFU/10^6^cells at visit 2 to 370 SFU/10^6^cells at visit 16, and 284 SFU/10^6^cells at visit 20 to 299 SFU/10^6^cells at visit 25), suggesting the increases observed, while modest, were vaccine-induced (Fig. S2). In summary, as previously reported^4^, VOR dosing had no effect on the HIV-specific T cell response. The AGS-004 vaccine induced T cell responses in *ex vivo* ELISpot in only 1 of 5 participants.

### Assessment of T cell and NK immune reactivity to AGS-004

Previously, we reported in both acute and chronically infected HIV participants on ART therapy, an increase in the CD28^+^/CD45RA^−^ CD8^+^ memory CTL response after AGS-004 administration^10,13^. Therefore, we assessed immune response in this way for participants receiving cycles of AGS-004 followed by VOR **(**Fig. 1). Baseline immune response was measured following VOR prior to AGS-004 dosing. Subsequent immune responses were measured at multiple timepoints during AGS-004 and VOR dosing, and at protocol completion.

**Figure 4:**
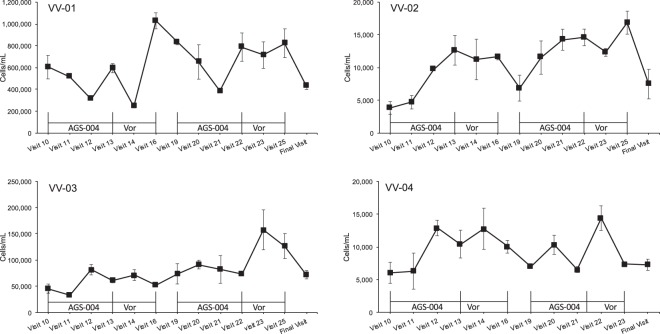
Immune response after *in vitro* stimulation with autologous DCs. Number of CD28^+^/CD45RA^−^ CD8^+^ memory CTLs proliferating (BrdU positive) or expressing CD107a, GrB, IFN-γ, IL-2, or TNF-α were determined at the indicated time points during cycles of combination therapy. The numbers of cells/mL were determined by adding the numbers of cells/mL measured for each individual functional marker. Values were derived from the average and standard deviation of triplicate cultures.

Numbers of CD28^+^/CD45RA^−^ CD8^+^ memory CTL determined after *in vitro* stimulation with autologous DCs during the first and second cycles of AGS-004 and Vorinostat are shown in Fig. 4. While increases in the numbers of CD28^+^/CD45RA^−^ CD8^+^ memory CTL could be detected at various timepoints after the first or second cycle of AGS-004 and/or during VOR treatment, only participants VV-02 and VV-03 had significantly increased numbers of CD28^+^/CD45RA^−^ CD8^+^ memory CTL which met the criteria for a positive immune response at visit 23. For participant VV-02 a positive response was recorded during both the first and second cycles of combined therapy. For VV-03 a positive response was recorded only during the second cycle of combined therapy. Individual functional marker responses within the total CD28^+^/CD45RA^−^ CD8^+^ memory CTL immune response for each subject are shown (Fig. S3).

**Figure 5:**
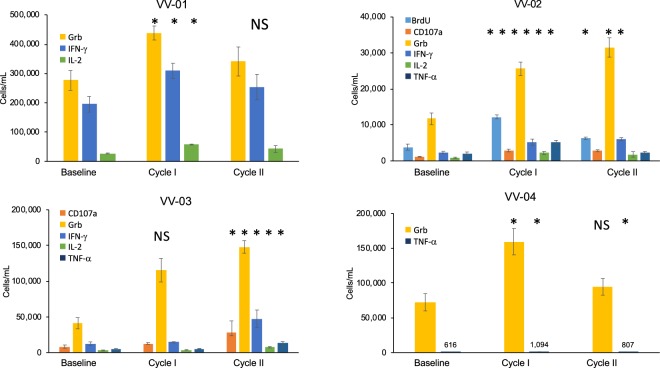
Total CD8^+^ T-cell functional immune responses after *in vitro* stimulation with autologous DCs. Functional CD8^+^ T-cells defined as proliferating (BrdU positive) or expressing CD107a, GrB, IFN-γ, IL-2, or TNF-α were determined at baseline and after cycle I and cycle II of combination therapy. Cycle I corresponds to Visit 16 and Cycle II to Visit 25. Only functional markers for which positive responses above baseline were observed are displayed in each chart legend. Values were derived from the average and standard deviation of triplicate cultures. Starred markers (*) indicate an increase in the numbers of functional CD8^+^ T-cell responses above baseline with p < 0.05; NS: p > 0.05.

To further understand an AGS-004-induced immune response, we measured the total numbers of CD8^+^ T cells with any functional response at baseline and after each cycle of combined therapy, where sufficient cells were available for analysis (VV01-VV04). This analysis was undertaken to gauge the overall functional CD8^+^ T cells response after *ex vivo* stimulation with autologous DCs. Overall there were statistically significant increases in CD8^+^ T-cells expressing granzyme B (Grb) *in vitro* for these participants (Fig. 5). The responses for participants VV-02 and VV-03 also became more multi-functional after cycle I and cycle II of combination therapy respectively with statistically significant increases in the numbers of CD8+ T cells secreting cytokines in combination with Grb.

**Figure 6:**
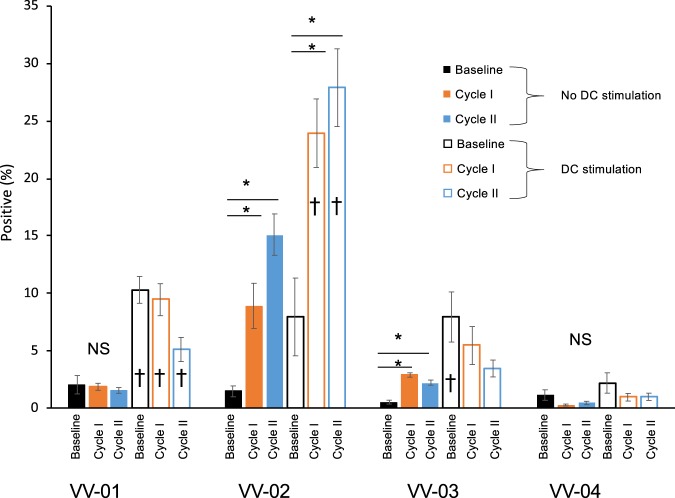
Determination of activated NK cells in PBMC co-cultures stimulated with autologous DCs. PBMCs at baseline, after cycle I, and after cycle II were cultured with or without autologous DCs for 6 days. Activated NK cells were identified as CD3^-^/CXCR4^-^/CD45RA^-^ cells expressing CD15, CD16, and GrB. Percentages of activated NK cells were determined from the average of triplicate cultures. ^†^Indicates p < 0.05 between no DC stimulation (filled bars) or with DC stimulation (open bars). *Indicates p < 0.05 cultures between no DC stimulation or with DC stimulation. NS: p > 0.05.

Recent observations have reported that the *in vivo* administration of histone deacetylase inhibitors do not impair natural killer cell function in HIV+ individuals^16^. Therefore, we also measured NK cell responses after a six-day PBMC co-culture with or without autologous DCs. NK cells were defined using the CD3^−^CD56^+^CD16^+^ phenotype, with negative expression for both CXCR4 and CD45RA. After co-culture, activated NK cells (defined by expression of Grb) were present in all participants, and the percentage of activated NK cells increased following the first and second cycles of VOR in participants VV-02 and VV-03. When autologous DCs were added to co-cultures, the percentage of activated NK cells was also increased for participants VV-01 and VV-02 (Fig. 6). There was no significant increase in the percentage of activated NK cells determined for subject VV-04.

### Viral Inhibition Assays

**Figure 7:**
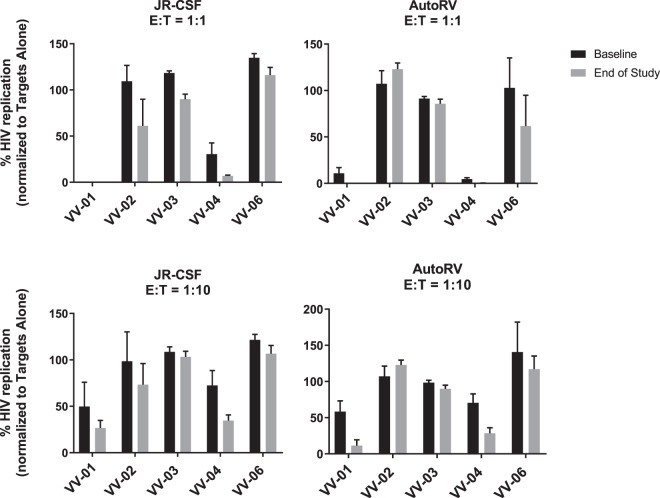
Viral inhibition assays using isolated CD8+ T cells from each of the participants before and after the clinical treatment. CD8 depleted PBMCs were stimulated with PHA and infected with either the viral strain JRCSF or autologous reservoir virus (AutoRV) and CD8+ T cells were added at an E:T ratio of 1:1 or 1:10. Viral replication was assessed by measuring HIV gag p24 in the supernatant after 6 days of culture. Results were normalized for each participant to the targets alone condition (100%). No significant statistical differences were found between the antiviral activity of CD8+ T cells isolated before and after treatment.

We have previously demonstrated the potential of various immunotherapies to augment HIV-specific effector responses, as measured by an *in vitro* autologous viral inhibition assay (VIA)^17,18^. To explore the possible correlations between *in vivo* responses in this pilot trial and the VIA, we assessed the antiviral activity of CD8^+^ T cells obtained after VOR and AGS-004 administration towards autologous participant CD4+ T cells super-infected with the laboratory strain JR-CSF or with HIV recovered and amplified from the individual’s resting CD4+ T cells (autologous reservoir virus, ARV). CD8 T cells from all participants except VV-01 and VV-04 had little antiviral activity at baseline in this assay. We observed a trend towards an increase in the anti-viral capacity of CD8 T cells post VOR and AGS-004 treatment for VV-02 against JRCSF, and for VV-01 and VV-04 against both JRCSF and autologous reservoir virus (Figs. 7 and S4). However overall, no significant statistical differences were found between the antiviral activity of CD8+ T cells isolated before and after treatment for all participants.

## Discussion

Currently antiretroviral therapy does not eliminate HIV-1 from latently-infected reservoirs^19–21^, and this remains the critical limitation to HIV eradication. Given a rapidly expanding number of HIV-infected individuals on lifelong ART worldwide, there is an urgent need for innovative therapies to control HIV-1 infection beyond what is currently possible with ART. We present the results of a pilot study combining a validated latency reversing agent with an immunotherapeutic shown to generate HIV-specific immune responses^13^.

Resting CD4^+^ lymphocytes comprise the largest and best characterized reservoir of HIV infection that persists despite ART^22^. Host cell molecular mechanisms that maintain the quiescence of HIV gene expression in infected resting CD4^+^ lymphocytes provide therapeutic targets within this largest and best-defined reservoir of HIV infection that persists despite ART. One of these latency mechanisms is the recruitment of histone deacetylases (HDACs) to the HIV long terminal repeat (LTR) promoter, mediating the formation of a repressive chromatin environment, a well-defined mechanism that inhibits LTR expression and HIV viral production. The relevance of this mechanism has been validated in numerous model systems and in the resting CD4^+^ T cells of ART-treated, aviremic individuals living with HIV^2^.

We and others have shown that HDAC inhibitors, such as VOR, can reverse HIV latency as measured by significant increase in cell-associated RNA expression^3,5–7^. In this study we administered VOR every 72 hours, as we had established this interval could repeatedly induce rca-RNA expression without significant clinical toxicity or immune dysfunction^4,23,24^.

In addition to latency-reversing agents, an enhanced and persistent HIV-1 specific immune response will be a required component of any cure strategy. Accordingly, this pilot study combined VOR administration with an HIV-1 specific immune therapy demonstrated activity in HIV-1 infected participants. The AGS-004 DC therapy is an individualized immunogen, and as generated from autologous PBMCs, addresses the inherent problem of HIV genetic diversity when using consensus HIV protein sequences as immunogens.

In a prior study, AGS-004 was safe and immunogenic in participants who initiated ART during acute HIV infection (AHI)^11^. DC therapy was administered to participants who initiated ART within 45 days of AHI and had HIV RNA < 50 c/ml for >6 months. Participants received 4 monthly doses of AGS-004 on ART. All six participants demonstrated positive immune responses defined as ≥2-fold increase from baseline in the number of CD28^+^/CD45RA^−^ CD8^+^ memory CTL that display effector function to the antigens contained in AGS-004, Vpr, Nef, Gag, and Rev. These responses were durable over 12 weeks.

Other DC-based HIV vaccines have been developed and undergone clinical testing, these include autologous DCs transfected with gag/nef mRNA^25^, DCs loaded with autologous HIV infected apoptotic cells^26^, DCs pulsed with heat-inactivated whole HIV^27^, and DCs loaded with HIV lipopeptides^28^. In most of these clinical trials a slight improvement in HIV specific T cell responses was observed in terms of activation, breadth and frequency of functional T cells. Unfortunately, virologic outcomes were limited upon treatment interruption, with only modest decreases in the viral load set point. However, a correlation between immune function enhancement and viral load reduction was observed. Finally, because of the large inter-individual variation observed in the vaccine response, it has been proposed that factors such as the host genetics might play an important role in immunotherapy outcomes^29,30^.

This pilot study in HIV-1-positive participants with durable viral suppression on stable ART sought to measure the potential of AGS-004 combined with Vorinostat to (1) stimulate expression of persistent proviral HIV from resting CD4^+^ T cells, (2) generate an HIV-specific immune response, and (3) when combined, clear persistent infection in HIV-infected participants in whom viral replication and spread is inhibited by uninterrupted ART.

12 participants enrolled in the study. One participant withdrew for personal reasons (relocation). Five were not advanced into the treatment steps of the protocol as no significant HIV RNA induction could be measured, most often due to low levels of RNA detected prior to cell treatment (<35 copies HIV RNA/million resting CD4 T cells). In only one participant, significant HIV RNA induction was measured *ex vivo*, but was not observed after a single oral dose of VOR, and so the participant did not advance to further treatment. In all five participants, *ex vivo* induction of HIV RNA expression was paralleled by induction following both a single dose of VOR, and by the second of two doses of VOR separated by 72 hours. Following this observation, in our other ongoing studies we now screen only for *in vivo* response using paired dosing.

Overall, we found the combination of AGS-004 DC therapy and VOR to be safe and well-tolerated. No study-treatment related adverse events greater than Grade I were observed. The most common Grade I events were mild, self-resolving injection site reactions related to AGS-004.

Assays to assess histone acetylation as an *in vivo* biomarker of VOR pharmacologic activity are time-consuming and qualitative. We employed a PCR-based assay described by Maxwell *et al*. (manuscript submitted) to assess the impact of VOR on host gene transcription, as a measure of host target engagement. The two host genes up-regulated (H1F0, IRGM) and two host genes that are down-regulated (PHF15 and PRDM10) were modulated as predicted upon repeated VOR dosing, suggesting that this assay will be useful in future clinical studies of HDAC inhibitors.

Unfortunately, these interventions had no measurable impact on the replication competent reservoir as measured by QVOA. We observed a significant decline of rca-RNA in two of 5 participants after the second cycle of combination therapy. This could reflect depletion of cells producing full-length HIV RNA, a substantial proportion of which might not have been replication-competent^31^ and might therefore not contribute to a decline of RCI. As the frequency of cells expressing HIV RNA is much larger than that of cells capable of producing replication-competent HIV^32^, a significant but modest decline of rca-RNA may not be measurable as a significant decline of RCI. Alternatively, this decline could have been driven by another unknown cause of assay variation, as the rca-RNA assay has not been extensively characterized in longitudinal studies^33^. Given the small sample size and lack of a control group, we cannot completely dismissed that some of the increase or decrease in caRNA is perhaps due to longitudinal variation in cRNA expression^34^. However, of note, VV-02 also had a decline in single-copy assay plasma viremia (SCA) from 4 copies/µl to <1.0 copies µl at all post-AGS-004 timepoints, but SCA levels were too low to assess in 3 other participants and were unaffected in a fourth (VV-06).

There are several limitations to this work including as mentioned earlier, the small sample size and lack of a control group for this pilot study. Further, it is possible that more of an effect would have been seen with this combined immunotherapeutic strategy in participants who initiated ART earlier during acute infection or with higher baseline CD4 counts; three of five participants in this study had a CD4 nadir <200. However, one participant (VV-01) who started ART during acute infection did not demonstrate an increased response compared to those starting ART during established HIV infection.

Another factor that should be taken into consideration is that while we have evidence from an *ex-vivo* model system that VOR can induce sufficient antigen in resting CD4+ T cells from ART-treated participants to allow clearance of HIV infected cells by autologous CD8 T cells^9^, this finding has not yet been validated *in vivo*. Furthermore, while we can infer that there is sustained antigen presentation by AGS-004 DCs based on prolonged IL-12 secretion as a result of the CD40L RNA co-electroporated along with the HIV RNA, we cannot completely dismiss the possibility that suboptimal HIV antigen presentation by DCs *in vivo* contribute to the limited success of this study. Indeed, these questions can only be fully addressed in an *in vivo* study such as this one.

In this limited pilot study, absent clear findings in primary virological endpoints, we sought to probe for correlations between immune effects and the limited virological effects seen in VV-01 and VV-02. However, augmentation of the several immune responses measured were not consistently seen in these two participants. In summary, the serial administration of VOR appeared to reverse HIV latency in this selected cohort without clinical toxicity. However, no substantial augmentation of anti-HIV immune function was uniformly seen in this cohort of durably treated individuals living with HIV. Surprisingly, the induction and magnitude of HIV-specific immune responses by AGS-004 was marginal and inconsistent across participants, in contrast to what has been reported for AGS-004 elsewhere^13^. The combination of AGS-004 DC therapy and VOR resulted in a decrease in cell-associated HIV RNA only in a minority of participants and had no substantial or uniform impact on the replication competent reservoir. Interestingly, depletion of the reservoir was not substantial even in the two participants with a measurable decrease in HIV rca-RNA, consistent with prior findings that most rca-RNA is replication defective. The need for the development of more potent latency reversal agents that robustly target the replication competent reservoir together with more efficacious immune interventions remains an important priority towards achieving an HIV cure.

## Materials and Methods

### Human subjects

This study was reviewed and approved by the University of North Carolina biomedical institutional review board, the NIAID Clinical Sciences Review Committee, and the Food and Drug Administration. This study, NCT02707900, was registered at Clinicaltrials.gov on 14 March 2016 and clinical trial monitoring performed by the NIAID. All research was performed in accordance with relevant US and institutional guidelines and regulations, and informed consent was obtained from all participants.

### Measurements of HDAC responsive genes

PBMC were collected immediately prior to the first VOR dose, 4 hours after the 5^th^ and 10^th^ VOR dose, and 7–10 days after cessation of dosing. RNA was extracted using the Qiagen RNeasy kit (Germantown, MD), and was quantified using the NanoDrop1000 (Thermo Fischer Scientific, Waltham MA). Three reactions of cDNA per sample, each with an RNA input of 500 ng, were prepared using the Maxima cDNA synthesis kit with dsDNase according to the manufacturer’s instructions (Thermo Fischer Scientific).

cDNA reactions were diluted 1:4 and subsequently used for qPCR in technical duplicates. 20 uL qPCR reactions consisted of 10 uL of QuantiTect Multiplex PCR NoROX Mastermix (Qiagen), 0.25 uL of AmpErase™ Uracil N-Glycosylase (Thermo Fischer Scientific), 2 uL of diluted cDNA, 400 nM or 1X primers, and 200 nM or 1X probes. Primers/probe sets for assessment of HDACi-modulated gene expression included previously validated primer/probe sets for H1F0, IRGM, and RPL27 from Integrated DNA Technologies^14^ or pre-designed 20X Taqman assays for PHF15 (Hs00959516) and PRDM10 (Hs00999748) from Thermo Fisher Scientific. qPCR cycling was conducted on the Bio-Rad C1000 Touch Thermal Cycler as follows: 50 °C × 2 min, 95 °C × 15 min, followed by 40 cycles of 94 °C × 1 min and 64 °C × 1 min. Cq values were determined using the automatic threshold analysis in the Bio-Rad CFX Maestro Software Version 1.1. Expression of HDACi-modulated host genes was assessed using the 2^−∆∆Ct^ method^35^. RPL27, a gene known to be non-responsive to HDACi stimulation, was used a reference gene^36^. For each round of vorinostat dosing in the study, fold change values were calculated relative to the baseline sample acquired immediately prior to dosing.

### Quantitative viral outgrowth assay (QVOA)

Lymphocytes were obtained by continuous-flow leukopheresis. Isolation of resting CD4^+^ T cells, recovery and quantification of replication competent virus was performed as described previously^3,37^. Briefly, approximately 50 million resting CD4^+^ T cells were plated in replicate limiting dilutions of 2.5 million (18 cultures), 0.5 million (6 cultures) and 0.1 million (6 cultures) cells per well, activated with PHA (Remel, Lenexa, KS) and a 5-fold excess of allogeneic irradiated PBMCs from a seronegative donor, and 60 U/ml IL-2 for 24 hours. Cultures were washed and co-cultivated with CD8-depleted PBMCs collected from selected HIV seronegative donors screened for adequate CCR5 expression. Culture supernatants were harvested on days 15 and 19 and assayed for virus production by p24 antigen capture ELISA (ABL, Rockville MD). Cultures were scored as positive if p24 was detected at day 15 and was increased in concentration at day 19. The number of resting CD4^+^T cells in infected units per million (IUPM) was estimated by a maximum likelihood method^3,38,39^.

### Measurement of cell-associated HIV DNA, RNA and low-level viremia

DNA was extracted from participant resting CD4 T cells using the Qiagen DNeasy® Blood & Tissue kit (Qiagen). Four replicates of 1 microgram of DNA was added to ddPCR™ Supermix for Probes (No dUTP) (Bio-Rad, Hercules, CA) with HindIII restriction enzyme for DNA digestion. Total HIV DNA was assessed using previously published primers and probe^40^ and standard ddPCR thermocycling conditions (Bio-Rad). Cell normalization was performed using two replicates of RPP30. Wells were merged together using the Bio-Rad Quantasoft Software and normalized to copies/million.

Changes induced in rca-RNA *in vivo* in response to VOR and AGS-004 dosing was measured as previously described with some minor modifications^3^. Immediately following leukapheresis, resting CD4^+^ T-cells were isolated and plated at 1 million cells/well, pelleted, snap frozen, and stored at −80 °C. Total RNA was isolated from 36 replicates of 1 million resting cells using the Magmax 96 Total RNA isolation kit (Life Technologies, Carlsbad, CA) following the manufacturer’s protocol. cDNA was synthesized in duplicate from DNase-treated, isolated RNA using the SuperScript® III First-Strand Synthesis SuperMix kit (Invitrogen, Carlsbad, CA) according to the manufacturer’s procedures. Reverse transcriptase was omitted from duplicate wells of each treatment condition and those wells served as controls for DNA contamination. Duplicate PCR amplification of duplicate cDNA was performed using the Biorad FX96 Real-Time PCR machine and previously published primers and probe^41^. A standard curve was generated for each PCR reaction as described previously^3^. Results of the four PCR replicates representing each of the original 36 pools of RNA were averaged and the standard deviation determined for each condition. Statistical significance was determined as described under the statistical analysis subheading.

To measure low level viremia, HIV-1 from plasma was isolated by ultracentrifugation, the resulting pellet was extracted as described in Cline *et al*.^42^. HIV gag RNA copy numbers were assessed using the qPCR assay described in Somsouk *et al*.^43^. HIV-1 viral loads are reported as copies/mL of plasma.

### AGS-004 Manufacturing and dosing

AGS-004 was manufactured at Argos Therapeutics, (Durham, NC) as previously described^10^. Monocytes were isolated from the leukocytes by elutriation and cultured in AIM-V media with 800 U/mL GM-CSF (Berlex Laboratories, Wayne NJ) and 1000 U/mL IL-4 (R&D Systems, Minneapolis MN) to generate immature DCs that are matured using 20 ng/mL TNF-α) (R&D Systems)/1000 U/mL IFN-γ (InterMune, Brisbane CA)/1 μg/mL PGE_2_ (Sigma, St. Louis MO). Mature DCs were electroporated with autologous pre-ART HIV RNA encoding Gag, Nef, Vpr and Rev and CD40L RNA using a post-maturation electroporation protocol described previously^10^. The final AGS-004 product was formulated as 1.4 × 10^7^ DC/0.7 mL in 80% autologous plasma, 10% dextrose (50% w/v) (Hospira Lake Forest IL), and 10% DMSO (Sigma) and cryopreserved in liquid nitrogen vapor phase. Each patient was treated with an autologous dose of AGS-004 supplied as a cryopreserved, single dose vial containing a minimum of 6 million autologous matured dendritic cells (target 1.2 × 10^7^ DCs) in 0.7 mL of suspension and is administered into a single lymph node basin as three intradermal injections of 0.2 mL each (0.6 mL total volume)^10^.

### Elispot assays

18-mer peptides overlapping by 10 amino acids were synthesized (Sigma-Genosys) to match the HIV Clade B consensus sequence and pooled by proteins in the AGS-004 vaccines Gag and Nef-Rev-Vpr and proteins not included in the vaccines, Pol, Env, Tat-Vif-Vpu. Peptide pools (quadruplicate) were pre-aliquotted into 96-well RB plates with media-only negative (6 replicates) and PHA positive control (duplicate) wells then stored at −80 °C. Cryopreserved PBMCs were thawed and rested O/N before being added to ELISpot plates (Merck, Millipore) at 4 × 10^5^ cells per well. All timepoints for each participant were run together. Antigens in the thawed peptide plates were mixed with 1:1 with PBMCs in the ELISpot plate to a final concentration of 2 μg/ml and incubated for 18–20 hours at 37 °C, 5% CO_2_. Coating, development (MabTech), and reading of ELISpot plates (AID Reader) has been described previously^44^. Positive T cell responses were defined as ≥12.5 SFU per million, >4 times the average of replicate background wells. Zero values were not accepted in any replicate of antigen-stimulated wells.

### Flow cytometry analysis of immune response

To determine immune responses by multi-color flow cytometry, PBMCs collected at the indicated timepoints (Fig. 1) for 4 of the 5 participants were cultured *in vitro* with autologous DCs product obtained from the manufacturing runs to measure HIV-specific T cell responses *in vitro* to the combination RNA antigen payload (GNVR) as described previously^10,45^. An insufficient amount of PBMC were obtained from participant VV-06 for this analysis.

Briefly, PBMCs were processed by Ficoll density gradient separation from whole blood draws and stored frozen. When all blood draws were collected from a participant, PBMCs were thawed and rested overnight in X-Vivo 15 supplemented with 10% Human AB serum. After overnight rest PBMCs were labeled with bromodeoxyuridine (BrdU) to track T-Cell proliferation and cultured with autologous DC targets and incubated at 37 °C for 6 days. On day 6, cultures were re-stimulated with autologous DCs and anti-CD107a antibody was added to each tube and incubated at 37 °C for 5 hours in the presence of Brefeldin A (BD Biosciences, Franklin Lakes NJ). After incubation, cells were stained for viability using a viability dye (Invitrogen, Carlsbad CA) followed by surface staining with specific antibodies for detection of CD28, CD45RA, CD3, and CD8 expression. After surface staining, cells were fixed with 4% BD Cytofix and stored overnight in BSA staining buffer at 4 °C. The following day, the cells were permeabilized and DNase treated for 1 hour at 37 °C using reagents included in the BrdU staining kit (BD Biosciences). Intracellular staining for IFN-γ, TNF-α, IL-2, Grb and BrdU were performed. After staining, cells were washed and diluted in 500 µl BSA buffer and transferred to a BD TruCount Tube (BD Biosciences) for acquisition on a BD LSRII cytometer. 400,000–600,000 events were collected per sample. Number of cells/ml was calculated using the following formula; (number of cellular events collected/number of beads collected) × (bead concentration)/collected volume) × 1000^45^.

The absolute number of viable CD8^+^ CD3^+^ T-cells positive for CD28 and negative for CD45RA and expressing any of the following six functional markers, IFN-γ, TNF-α, IL-2, GrB, CD107a, or the expression of the proliferation marker, BrdU were identified to the total HIV antigen payload. To determine the total number of functional CD28^+^/CD45RA^−^ CD8^+^ memory CTLs, the numbers of CD28^+^/CD45RA^−^ CD8^+^ memory CTLs having any functional activity were added together to determine absolute cells/mL. Per protocol, a positive immune response to AGS-004 was defined as >2-fold increase (plus 3 times STD) over baseline in the absolute number of CD28^+^/CD45RA^−^ CD8^+^ memory CTLs (cells/mL) exhibiting at least one effector function defined by the expression of IFN-γ, TNF-α, IL-2, GrB, CD107a, or proliferation. Baseline was defined as Visit 10 to designate the time prior to administration of AGS-004.

For detection of NK cells, frozen PBMCs were thawed and rested overnight and then cultured for 6 days with or without autologous DCs. After 6 days of culture cells were surface stained with antibodies to the following markers: CD3, CD4, CD8, CD25, CD45RA, CD16, CD56, and CxCR4 followed by staining for viability using Aqua Live/Dead Fixable Dye. Cells were washed and Transcription Factor Buffer set (BD Biosciences) was used to fix the cells. Following overnight storage at 4 °C in FBS stain buffer, cells were permeabilized by washing in Perm Wash buffer. Human IgG FC block was added to prevent non-specific antibody binding. After washing intracellular antibody to Grb was added. Cells were washed and acquired in BD True Count tubes for acquisition on a BD LSRII cytometer using FACSDiva software and 400,000–600,000 events were collected per sample. Data from acquired samples were analyzed using Flowjo software versions 9.7.6 (Ashland OR). Insufficient PBMC were collected from participant VV-04 at visit 25 for analysis so visit 23 was used for an analysis after cycle II.

### Viral inhibition assay

Viral inhibition assay was performed as previously described^17^. CD8^+^ T-cells were isolated from PBMCs by positive selection (EasySep human CD8^+^ Selection Kit, Stem Cell, Vancouver, BC). CD8-depleted PBMCs were first activated with 4 μg/mL of PHA (Remel, Lenexa, KS) and 60 U/mL of IL-2, and then infected by inoculation at 1200 × g for 90 minutes with either JR-CSF or autologous reservoir virus (AR) at a MOI of 0.01. AR virus was obtained from pooled supernatants of replicate wells from outgrowth assays of resting CD4^+^ T-cells from each patient. Fifty-thousand (5 × 10^4^) targets/well were co-cultured with CD8^+^ T cells in triplicate at the indicated effector to target (E:T) ratio in 0.2 m of cIMDM media supplemented with 10% FBS, 1% Penicillin/Streptomycin and 5 U/mL IL-2. P24 concentration in supernatant was assayed on day 6 by p24 ELISA (ABL, Rockville, MD).

### Statistical analysis

The Wilcoxon Two-Sample test was used for statistical analysis of rca-RNA of pre- and post-VOR+ AGS-004 exposure groups. For viral inhibition assays, the Wilcoxon-Signed Rank test was used for statistical assessment of the effect of AGS-004 on the anti-viral function of CD8+ T cells.

CD28^+^/CD45RA^−^ CD8^+^ memory CTL responses at baseline and after AGS-004 administration were compared statistically using a 2-tailed Student *t*-TEST. Positive responses were determined by subtracting two times the total number of functional CD28^+^/CD45RA^−^ CD8^+^ memory CTLs at baseline, from the total number of functional CD28^+^/CD45RA^−^ CD8^+^ memory CTLs measured in response to *in vitro* stimulation with DC encoding the HIV antigens. Finally, the percentage of activated NK cells at baseline and after AGS-004 administration were compared statistically using a 2-tailed Student *t* test.

## Supplementary information

Supplementary Tables S1-S2 and Figures S1-S4.

## Data Availability

All data is available in the main text or the supplementary materials.
