# Supplementary material for: Assessing the impact of AGS-004, a dendritic cell-based immunotherapy, and vorinostat on persistent HIV-1 Infection
Source: Sci Rep. 2020 Mar 20;10:5134. doi: 10.1038/s41598-020-61878-3 (PMC7083965; doi:10.1038/s41598-020-61878-3)
Supplement: Supplementary file 1 — Supplementary Tables S1-S2 and Figures S1-S4. [file 41598_2020_61878_MOESM1_ESM.pdf]

Supplementary material for:
Assessing the impact of AGS-004, a dendritic cell-based immunotherapy, and vorinostat on persistent HIV-1 Infection

Cynthia L. Gay[1,2], Joann D. Kuruc[1,2], Shane D. Falcinelli[1,3], Joanna A. Warren[1,3], Sarah A. Reifeis[4] , Jennifer L. Kirchherr[1] , Katherine S. James[1], Morgan G. Dewey[1], Alyson Helms[1,2], Brigitte Allard[1], Erin Stuelke[1], Alicia Gamble[7], Ana Plachco[7], Robert J. Gorelick[6], Joseph J. Eron[1,2,5], Michael Hudgens[4], Carolina Garrido[1,2], Nilu Goonetilleke[1,3], Mark A. DeBenedette[7], Irina Y. Tcherepanova[7], Charles A. Nicolette[7], Nancie M. Archin[1,2], and David M. Margolis[1,2,3,5]*

University of North Carolina HIV Cure Center[1], UNC Institute of Global Health and Infectious Diseases, Departments of Medicine[2] and Microbiology and Immunology[3], UNC Chapel Hill School of Medicine, Department of Biostatistics[4] and Epidemiology[5], UNC Chapel Hill School of Public Health, University of North Carolina at Chapel Hill, Chapel Hill, North Carolina, AIDS and Cancer Virus Program[6], Frederick National Laboratory for Cancer Research, Frederick, MD, and Argos Therapeutics[7], Durham, North Carolina, USA.

*Correspondence should be addressed to: David Margolis MD
Phone (919)-966-6388
dmargo@med.unc.edu

Table S1. Effect of AGS-004 and VOR on residual viremia (HIV copies/ml)

PID	Baseline	Cycle I*	Cycle II	Post-study
VV-01	0.4	0.49; 0.63	1.1	0.34
VV-02	4.3	0.42; 0.34	BLD	0.29
VV-03	0.15	BLD; 0.14	0.55	0.5
VV-04	0.2	0.55; 0.18	0.47	0.45
VV-06	3	12; 84[Ψ]	0.17	19

BLD: below the limit of detection

** Visits 14 and 17 respectively*

Ψ Documented episode of blips

Table S2. Participant resting cell infection (infected cells/million resting CD4+ T cells with 95% Confidence Interval)

Participant ID	Baseline	After 2nd cycle
VV-01	0.315 (0.187, 0.525)	0.258 (0.138, 0.447)
VV-02	1.035 (0.612, 2.256)	0.591 (0.323, 1.106)
VV-03	0.544 (0.313, 0.947)	0.527 (0.304, 0.927)
VV-04	1.042 (0.648, 2.011)	1.260 (0.780, 2.512)
VV-06	0.326 (0.146, 0.672)	0.390 (0.217, 0.697)

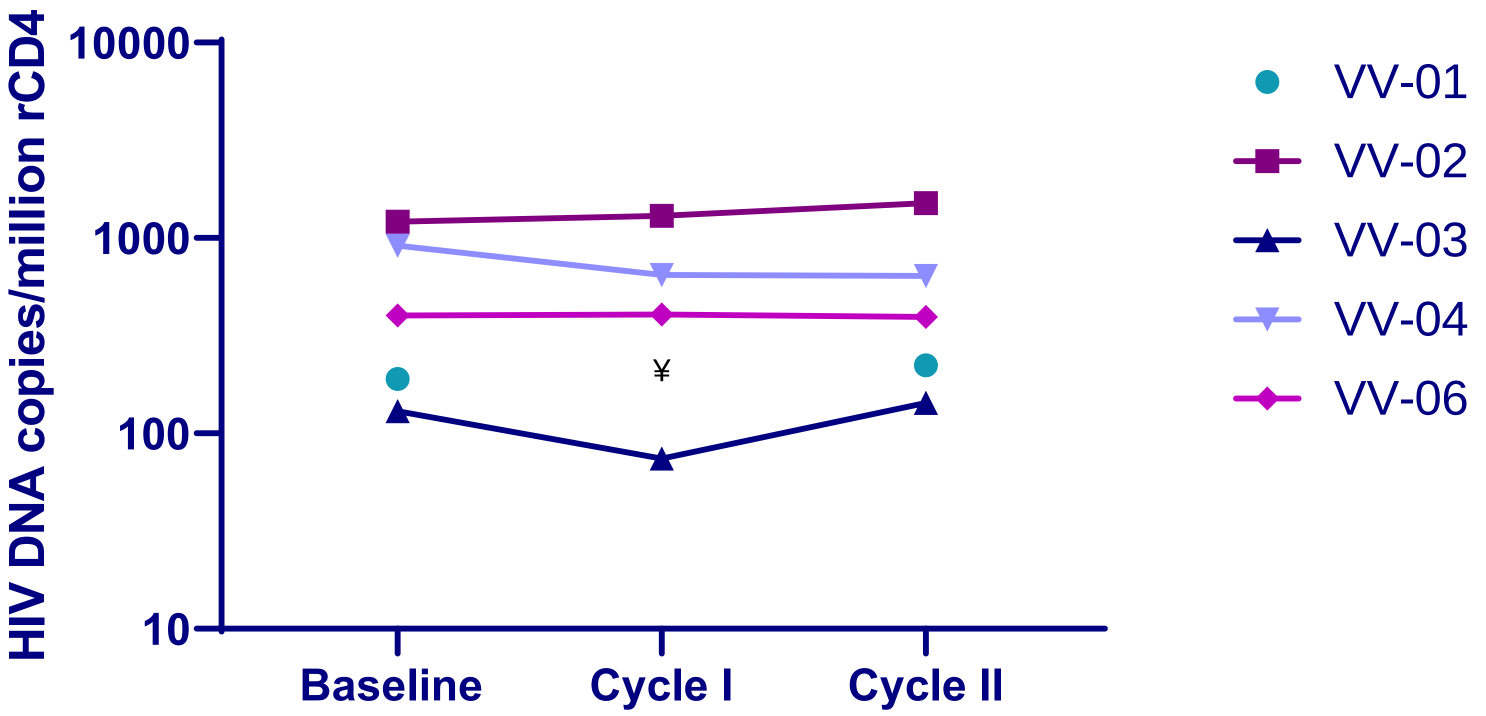

Supplementary Figure 1. No significant changes in total HIV DNA after AGS-004 and VOR administration. Cell associated HIV DNA was measured in 5 million resting CD4+ T cells for each time point shown by ddPCR. The gene RPP30 was used as a reference to normalized to copies/million. ¥, samples for this time point not available.

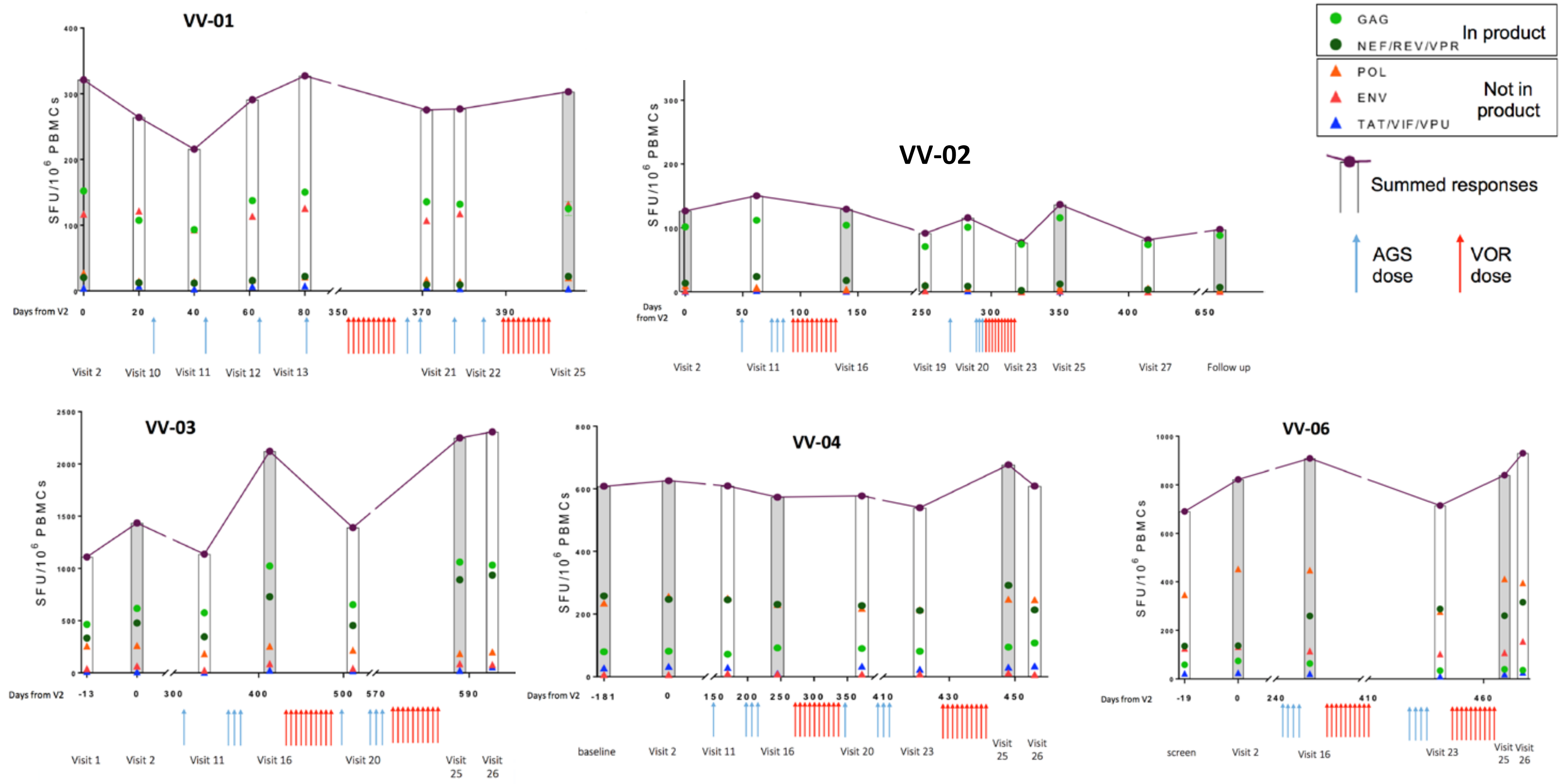

Supplementary Figure 2. T cell response to HIV proteins before and after VOR and AGS-004 dosing. Cryopreserved PBMCs were thawed, rested overnight, added to ELISpot plates containing peptide pools representing proteins within the AGS-004 vaccine (Gag, Nef-Rev-Vpr), and others not in the vaccine (Env, Pol, Tat-Vif-Vpu and incubated overnight. Media and PHA were used as negative and positive controls respectively. Detection and enumeration of IFN-y producing cells in response to peptide stimulation was quantitated by ELISpot assay. Positive T cell responses were defined as ≥12.5 SFU per million, > 4 times the average of replicate background wells. The AGS-004 vaccine induced T cell responses in *ex vivo* ELISpot in participant VV-03 only. Grey bars indicate PBMC from leukapheresis products; white bars indicate PBMC from whole blood.

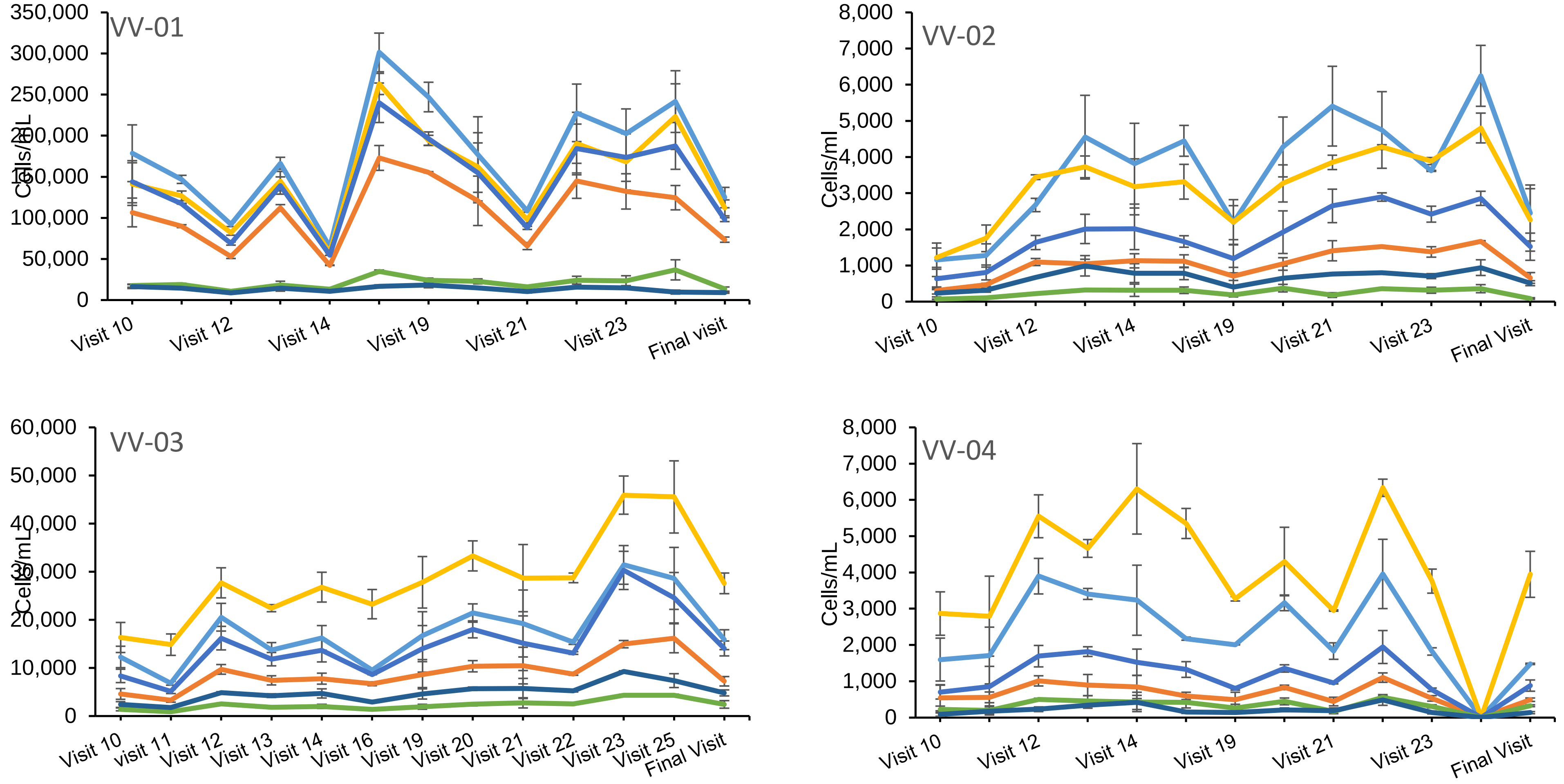

Supplementary Figure 3. Proliferative and cytokine responses in $CD28^{+}$/$CD45RA^{-}$ $CD8^{+}$ Memory CTLs during combination therapy. PBMCs, from whole blood collected at the indicated visits, were cultured with autologous DCs. The number (cells/mL) of proliferating cells or cells expressing CD107a, GrB, IFN-g, Il-2, or TNF-α were determined from $CD28^{+}$/$CD45RA^{-}$ $CD8^{+}$ T-cell subset. Each functional marker is represented by the following symbols BrdU ■, CD107a ■, Grb ■, IFN-γ ■, IL-2 ■, and TNF-α ■. Cells/mL values were determined by the average of triplicate cultures. Visit 10 designates the time prior to administration of AGS-004.

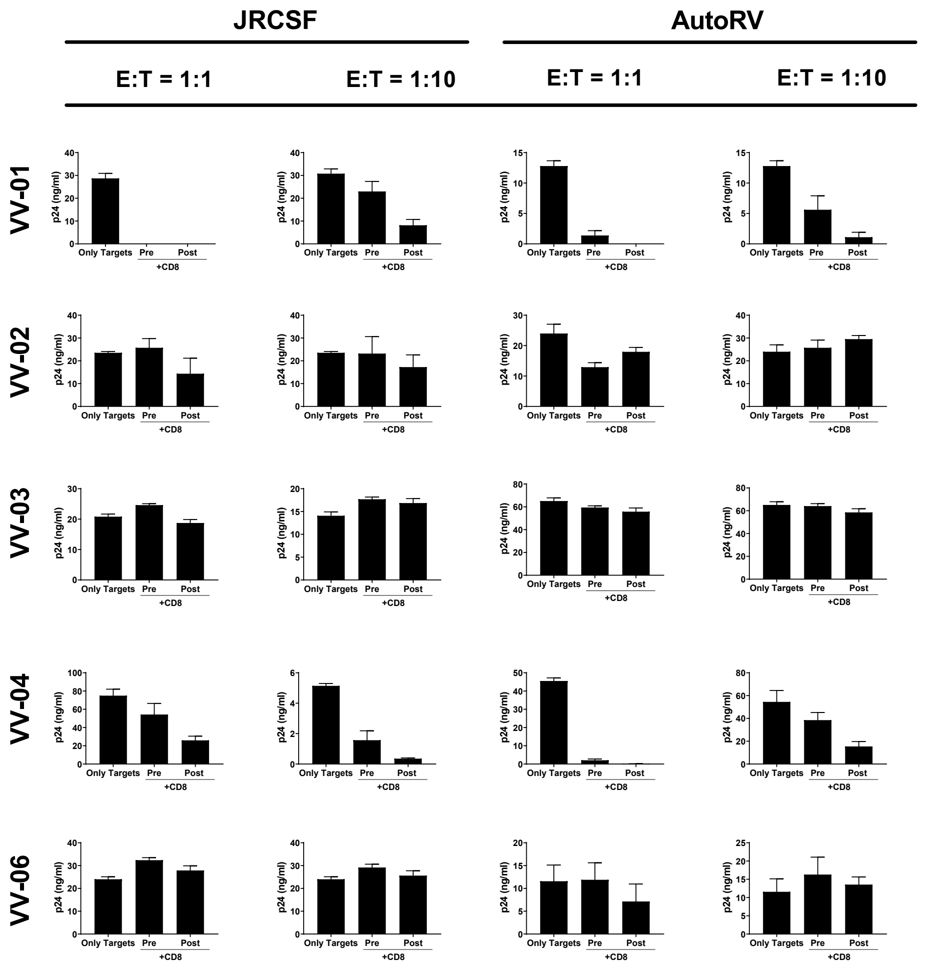

Supplemental Figure 4. Non-normalized data from viral inhibition assays using isolated CD8+T cells from each of the participants before and after the clinical treatment. CD8 depleted PBMCs were stimulated with PHA and infected with either the viral strain JRCSF or autologous reservoir virus (AutoRV), and then CD8+T cells were added at E:T ratio of 1:1 or 1:10. Viral replication was assessed in the supernatant after 6 days of culture by HIV gag p24 ELISA.
